# Patients’ Radiation Shielding in Interventional Radiology Settings: A Systematic Review

**DOI:** 10.7759/cureus.16870

**Published:** 2021-08-04

**Authors:** Mohamed T El-Diasty, Ahmed A Olfat, Ahmad S Mufti, Ahmed R Alqurashi, Mohammed J Alghamdi

**Affiliations:** 1 Department of Radiology, King Abdulaziz University Hospital, Jeddah, SAU; 2 Department of Radiology, King Abdullah Medical City, Mecca, SAU

**Keywords:** interventional, radiology, transjugular intrahepatic portosystemic shunt, hepatic arterial chemoembolization, shielding

## Abstract

As a result of the increasing risk of developing radiation-related complications, many approaches aimed at reducing this risk and enhancing the outcomes of the patient, doctor or device operator have been developed. In this systematic review, we aim to discuss previous investigations that studied patient shielding or protection within the context of selected interventional radiology procedures. We included original studies that used K_a,r, _and P_KA_ for the assessment of the outcomes of two procedures: transjugular intrahepatic portosystemic shunt creation (TIPS) and hepatic arterial chemoembolization (HAE). A thorough search strategy was conducted on relevant databases to identify all relevant studies. We included 13 investigations, including 12 cross-sectional studies and one randomized controlled trial. Significant diversity was found among all these studies in terms of the used modalities, which made them hard to compare. However, almost all studies agreed that using novel imaging and interventional modalities is useful when obtaining better outcomes and reducing patient radiation exposure. The use of ultrasound-guided procedures and providing adequate lead curtains has also been recommended by the identified studies in order to minimize the frequency of radiation exposure. The reported K_a,r, _and P_KA _were also variable between studies and were discussed within this study. Our findings indicate that unified guidelines for patient radiation shielding should be urgently investigated.

## Introduction and background

As a result of increasing medical advances in interventional radiology techniques and the reported benefits of various related fluoroscopic guided procedures, there can be prolonged procedures exposing the patient and staff to higher amounts of radiation with potential short- and long-term effects. However, this has been met by concern and caution as the increased prevalence of these procedures subsequently increases the risks associated with radiation exposure. Although it is now clear that interventional radiology plays an important role in the management of many diseases and fewer reported complications than those resulting from invasive surgical procedures, previous studies have demonstrated that interventional radiological procedures may be associated with increased side effects including hair loss and other tissue-reactive abnormalities that are secondary to the increased exposure to radiation [1-5].

There are many precautions and steps that can protect against complications related to interventional radiological procedures. In particular, which require the integration of novel modalities to reduce the maximum skin dose during radiation exposure (Dskin, max) [6,7]. Additionally, previous studies have demonstrated that an estimation of Dskin, max can be undertaken using various modalities including metal-oxide-semiconductor field-effect transistors, four photoluminescence sensors attached to the back of the patients, and wireless dosimeter modalities [8-11]. Although this approach has been previously reported to have various benefits when estimating the real-time data of radiation exposure, subsequent evidence indicates that it had some limitations which restrict its benefits to specific institutions [12,13]. As a result of these limitations, previous investigations reported the efficacy of both air kerma at the patient entrance reference point (Ka,r), and the directly assessed Dskin, max of the exposed patients [14-16]. Moreover, it has been indicated that both modalities have a significant correlating factor, and estimating the value of any of these modalities can obtain that of the other [17-19].

As a result of the increased risk of developing radiation-related complications, many suggestions have been made to reduce the risk and enhance the outcomes for the patient, doctor or device operator. Some studies reviewed here reported a potential reduction of radiation risk in their population as measured by Ka,r, and air kerma-area product (PKA) [20]. As such, in this systematic review we aim to address those studies that have assessed patient shielding or protection in selected interventional radiology procedures, as measured by the aforementioned two parameters.

## Review

Methods

Definition of Outcomes and Inclusion Criteria

In this systematic review, we aim to discuss those previous investigations that have studied patient shielding or protection in the setting of selected interventional radiology procedures. We have included original studies that encompass hepatic-related procedures as these were commonly found in the literature with sufficient population and reporting of outcomes. Moreover, it has been identified that these procedures imply that patients are more frequently exposed to irradiation than other specialized interventional procedures. Accordingly, we only included studies that were based on creating transjugular intrahepatic portosystemic shunt (TIPS) and hepatic arterial chemoembolization (HAE) procedures.

Studies that used the K_a,r_, and P_KA_ were utilized for the assessment of these outcomes. K_a,r_ usually refers to the initial kinetic energy that was released per total mass of the released air per the interventional reference point. This has been previously determined by the International Electrotechnical Commission to be 60601-2-43, and is measured by Gy. The location of the Ka,r has been previously referred to as the center of the X-ray bundle and 15 cm away from the C-arm isocenter of the same bundle, which is usually located within or towards the focal spot. In clinical settings, Ka,r can be used to assess whether the threshold of radiation skin exposure has been exceeded, which can prevent the development of complications. It can also classify the severity of the clinical outcomes based on the dose of radiation. Previous studies have demonstrated that when excluding fluoroscopically guided interventions, Ka,r is the best modality to assess skin radiation exposure. Conversely, PKA or dose area product (DAP) is measured by Gy per cm^2^ and usually refers to the plane where the area of the X-ray beam and the cross-sectional area of the Ka,r has been identified. In clinical settings, PKA can be used for the estimation of stochastic risk as it can estimate the total effective dose of radiation and the total amount of energy consumed. Stochastic effects are important and can cause serious complications and affect the patient’s quality of care. All of the included articles in this review have assessed their outcomes on a patient population.

Articles that did not assess the aforementioned outcomes or identified procedures were excluded. Articles that were not original or did not investigate the outcomes or the effect of dose reduction on a patient population were also excluded, as were theses, protocols, abstract-only articles, and non-English studies.

Search Strategy

Our search strategy was carefully designed to identify all related citations, and the study members also identified relevant articles to assist with locating all relevant databases. The search terms were modified when needed. The search strategy was confined to articles that were published post-2011 and any articles published before this period were excluded. We searched PubMed, Scopus, Google Scholar, Embase, Cochrane library, Virtual Health Library, and Web of Science. Review articles were also used to identify any relevant citations that could have been missed by the main electronic search strategy.

Article Screening

This step was undertaken by all study authors, which required the integration of all efforts to obtain the best outcomes. When the search was complete, the study leader exported all the results, which had been agreed to by all members, to a unified endnote library designed to exclude the duplicated studies identified by the different database searches. The remaining articles were then exported into an excel datasheet for criteria-based screening. Title and abstract screening were followed by full-text downloading and each screening was undertaken by at least two researchers for each article, and each of these researchers was blind to the results of the other to restrict cheating and collusion. Discussions between the researchers and supervision by a senior leader were also utilized whenever a conflict was observed among the screened articles. All steps were undertaken per the Preferred Reporting Items for Systematic Reviews and Meta-Analyses (PRISMA) guidelines [21].

Data Extraction and Quality Assessment

After the final check of the screened articles, a suitable extraction spreadsheet was created to contain all the relevant information that could help us report our outcomes. The spreadsheet consisted of three parts. First part included a section for baseline characteristics such as author name, country, DOI, sample size, study design, gender, and age. The second part consisted of the outcomes such as the type of the procedure, the applied radiation interventional modality, and the estimated P_KA_, DAP, and K_a,r_. Lastly, the last spreadsheet contained the quality assessment. This section was conducted using the modified Newcastle-Ottawa scale (NOS) for cross-sectional studies [22]. The scale is composed of three main domains assessing the quality of assessment, reporting, and compatibility. Studies were classified based on their assessed grades and quality standards.

Results

Study Selection

**Figure 1:**
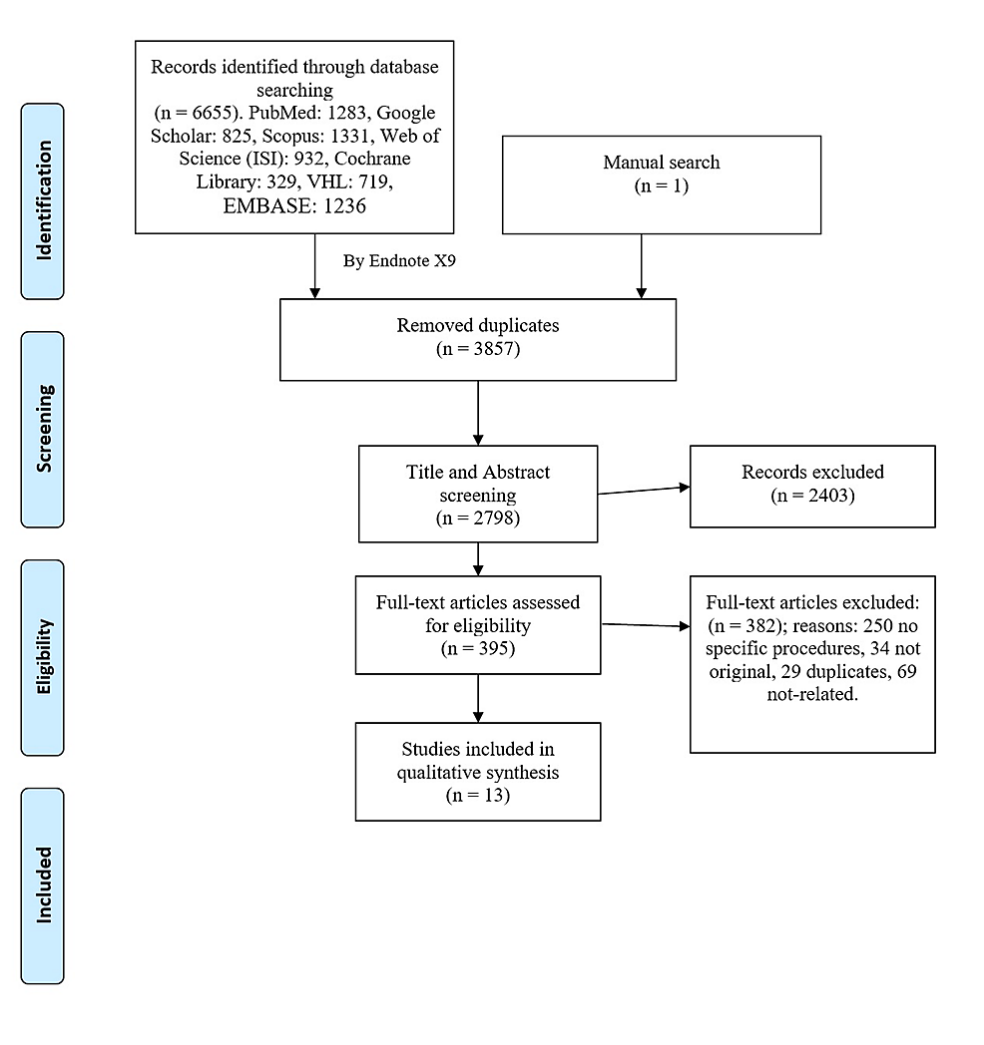
PRISMA flow chart for the selection process to include the relevant studies. PRISMA: Preferred Reporting Items for Systematic Reviews and Meta-Analyses.

A summary of this step can be seen in Figure 1 flow chart. In brief, we exported a total of 6,655 citations to the endnote library, which was then used to exclude duplicates. The final screening of the remaining articles identified 13 related studies that met our criteria.

Risk of Bias

None of the included studies in this review had a non-satisfactory quality of bias as assessed by our authors. All studies showed favorable qualities as the results ranged between good and satisfactory markings according to the grades of these studies (graded from 0 to 10). The specific themes and grading of domains of the web of science (WOS) scale are presented in Table 1. Only one randomized controlled trial was included and our assessment indicated that the study had a low risk of bias according to Cochrane’s collaboration tool for assessment of bias in randomized studies.

**Table 1:** Quality assessment of the included studies by the modified Newcastle-Ottawa scale (NOS).

Author	Year	Selection				Comparability	Outcome		Total score	Quality
		Representativeness of the sample	Sample size	Non respondents	Ascertainment of the exposure	The subjects in different outcome groups are comparable	Assessment of outcome	Statistical analysis		
Khoury et al. [23]	2015	+	+	+	+	+	+	+	7	Good
Dave et al. [24]	2016	+	+		+	+	+	+	6	Satisfactory
Kothary et al. [25]	2011	+	+	+	+	++	+	+	8	Good
Livingstone et al. [26]	2011	+	+	+		+	+	+	6	Satisfactory
Ruiz-cruces et al. [34]	2016	+	+		+	+	+	+	6	Satisfactory
Miraglia et al. [27]	2015	+	+	+	++	+	+	+	8	Good
Panick et al. [28]	2018	+	+	+	+	++	+	+	8	Good
Wen et al. [29]	2015	+	+	+	+	+	+	+	7	Good
Spink et al. [30]	2017	+	+		+	+	+	+	6	Satisfactory
Bundy et al. [31]	2018	+	+	+	+	++	+	+	8	Good
Tavare et al. [32]	2017	+			+	+	+	+	5	Satisfactory
Kwak et al. [33]	2020	+	+	+	+	+	+	+	7	Good

Baseline Characteristics

This systematic review was comprised of a total of 12 relevant cross-sectional studies and one randomized controlled trial. All articles contributed to the investigation of our declared outcomes and helped us assess our final evidence. Among these studies, six were from the United States, and one each from Brazil, India, China, Italy, Germany, Spain, and the United Kingdom. All of the studies were prospective bar two studies that were based on a retrospective analysis. The number of performed procedures in the interventional radiology units was also variable, ranging between 11 and 212 procedures. Moreover, six studies included patients that underwent TIPS procedures only, another six included patients who underwent only HAE procedures, and only one study included both procedures in their population. Other baseline characteristics and a summary of the outcomes can be seen in Table 2.

**Table 2:** Baseline characteristics and summary of the outcomes of the included studies.

Reference	Year	Country	Study design	Data collection	Sample size	BMI	Mean age	Procedure	Number of procedures	DSAs number	PKA (Gycm2 )	Ka,r (Gy)	Author conclusion	
														Khoury et al. [23]	2015	Brazil	Cross-sectional	Prospective	55	-	62.2	HAE	55	250	267.49, 403.83, 479.74	1.8	Providing lead curtains and insulators can reduce the frequency of radiation exposure and enhance the outcomes.	
Dave et al. [24]	2016	United States	Cross-sectional	Prospective	26	-	-	HAE	33	113	134.03	279	ClarityIQ can reduce the complications of radiation and minimize exposure.	
Kothary et al. [25]	2011	United States	Cross-sectional	Prospective	87	28.2	44-86	HAE	72	-	184.2	626.4	C-arm CT is associated with increased stochastic risk and reduced deterministic risk than DSA and can be enhanced by experience	
Livingstone et al. [26]	2011	India	Cross-sectional	Prospective	19	-	-	TIPS	19	-	63.86	-	Ultrasound guidance can achieve better protection outcomes	
Ruiz-cruces et al. [34]	2016	Spain	Cross-sectional	Prospective	1649	-	-	HAE	138	-	216.1	-	Novel technological and imaging processing modalities can achieve patient protection	
Miraglia et al. [27]	2015	Italy	Cross-sectional	Prospective	347	27.7	56	TIPS	88	-	360	-	Ultrasound guidance can achieve better protection outcomes	
Schernthaner et al. [28]	2015	United States	RCT	Prospective	66	-	-	HAE	78	-	132.9 and 395.8	0.5 and 2.1	Optimized acquisition parameters and improved imaging modalities can reduce radiation exposure.	
Panick et al. [20]	2018	United States	Cross-sectional	Prospective	134	30.3		TIPS	14	-	191.8	0.76	Fluoroscopy guidance reduced the frequency of radiation exposure and enhanced the outcomes	
Panick et al. [20]						28		HAE	60	243	170.4	1.04		
Wen et al. [29]	2015	China	Cross-sectional	Prospective	50	23	57	HAE	50	173	77.3	0.17	Novel technological and imaging processing modalities can achieve patient protection	
Spink et al. [30]	2017	Germany	Cross-sectional	Prospective	108	25	57	TIPS	54	-	173.3	0.7	Upgrading imaging technologies significantly reduce the frequency of radiation exposure	
Bundy et al. [31]	2018	United States	Cross-sectional	Prospective	4784	-	55	TIPS	120	-	429.2	2.002	Upgrading imaging technologies significantly reduce the frequency of radiation exposure	
Tavare et al. [32]	2017	United Kingdom	Cross-sectional	Retrospective	212	-	-	TIPS	212	-	40.3	404.3	Ultrasound guidance can achieve better protection outcomes	
Kwak et al. [33]	2020	United States	Cross-sectional	Retrospective	11	-	-	TIPS	11	-	8.4	29.5	Ultrasound guidance can achieve better protection outcomes	

Discussion

TIPS

Many of the studies included in this review assessed the efficacy of their novel approaches for reducing the dose of radiation, minimizing the risk of developing radiation exposure, and achieving patient protection. The investigation by Livingstone et al. [26] reported that significant radiation protection was obtained in their population after the introduction of ultrasound-guided modalities for their patients resulted in lower estimated total DAP levels. This was also supported by the Miraglia et al. [27], Tavare et al. [32], and Zhang et al. [33] studies, which reported that DAP levels were significantly lower in patients who underwent ultrasound-guided procedures. A previous investigation by Panick et al. [20] also evaluated the efficacy of a new modality (Discovery IGS740, GE Healthcare) for obtaining better outcomes in multiple hepatic procedures. The authors reported a significant reduction in both the P_KA_ and K_a,r_ levels for TIPS procedures, as noticed in the novel room when compared to the other previous modalities. The AlluraClarity was also developed by Spink et al. [30] in 2017 and demonstrated a significant reduction in radiation parameters without interfering with the quality of the outcomes. Bundy et al. [31] also compared their approach to the RAD-IR study conducted in 2003 and reported that the previously proposed reference levels for DAP and time spent on fluoroscopy were identified among their recommended procedures, including TIPS and hepatic chemoembolization.

HAE

The effect of patient protection and radiation dose reduction in HAE procedures was also investigated by many of the selected articles. The previous study by Khoury et al. [23] reported the radiation doses for patients treated across three hospitals. They reported differences between the total P_KA_ levels (267.49, 403.83, and 479.74 Gy cm^2^) between the three hospitals, who each used a different imaging modality. Accordingly, the authors suggested that interventional approaches such as lead shields should be adequately installed in the hospitals to avoid possible skin-related complications. However, these were not widely available in all healthcare settings, and when available, they were not widely used by the attending physicians. Another study by Dave et al. [24] reported that the P_KA_ and K_a,r_ levels were significantly lower when the new imaging modality Clarity IQ was installed in their settings, however, the imaging quality for this technique was relatively lower than other modalities that did not feature the technique. The study by Kothary et al. [25] reported that the application of a C-arm CT was associated with more frequent stochastic risk, as expressed by DAP, and increased reduction in the deterministic risk than the digital subtraction angiography procedure. However, they also reported that the quality of protection with the C-arm modality can be enhanced by increasingly experienced physicians. Positive instances of C-arm imaging technique use were reported by Schernthaner et al. [28], who demonstrated that the modality was able to reduce the time of the procedure without interfering with the quality of the obtained images. The Ruiz-Cruces et al. [34] study stratified the diagnostic reference levels for hepatic chemoembolization procedures based on the complexity of the procedure. They reported that the P_KA _levels for the simple, medium and complex procedures should not exceed 170, 303, and 881 Gy cm^2^. Therefore, the complexity of the procedure must be estimated in order to determine the optimum dose of radiation. The new suite developed by Panick et al. [20] also managed to obtain better outcomes and protection in HAE procedures when compared to previously studied modalities. Similarly, a new system was also developed by Wen et al. [29] which demonstrated less radiation exposure with a maintained imaging quality, as a result of the reduced time and energy of exposure.

Our results may be limited to the designs of the included studies, which lacked proper randomization of patients and adequate sample sizes. Moreover, we could not formulate adequate analyses to obtain the most favorable and efficacious modalities for reducing exposure to radiation during radiological interventions, due to the heterogeneity among the included studies.

## Conclusions

Our systematic review discussed current approaches to achieving patient radiation protection. We found great diversity among the included studies and almost all developed a novel strategy for achieving patient protection. However, most studies agreed on using combination of imaging-processing techniques and ultrasound-guided interventions for enhancing outcomes, thus reducing radiation exposure and decreasing the frequency of potential complications. Unified guidelines for these processes should be urgently considered via further investigations.
